# BEX1 and BEX4 Induce GBM Progression through Regulation of Actin Polymerization and Activation of YAP/TAZ Signaling

**DOI:** 10.3390/ijms22189845

**Published:** 2021-09-11

**Authors:** Sungmin Lee, Hyunkoo Kang, Eunguk Shin, Jaewan Jeon, HyeSook Youn, BuHyun Youn

**Affiliations:** 1 Department of Integrated Biological Science, Pusan National University, Busan 46241, Korea; smlee1048@gmail.com (S.L.); kanghk94@gmail.com (H.K.); egshin94@gmail.com (E.S.); 2 Department of Radiation Oncology, Haeundae Paik Hospital, Inje University College of Medicine, Busan 48108, Korea; jjw1066@paik.ac.kr; 3 Department of Integrative Bioscience and Biotechnology, Sejong University, Seoul 05006, Korea; hsyoun@sejong.ac.kr; 4 Department of Biological Sciences, Pusan National University, Busan 46241, Korea

**Keywords:** glioblastoma, actin polymerization, BEX1, BEX4, latrunculin B

## Abstract

GBM is a high-grade cancer that originates from glial cells and has a poor prognosis. Although a combination of surgery, radiotherapy, and chemotherapy is prescribed to patients, GBM is highly resistant to therapies, and surviving cells show increased aggressiveness. In this study, we investigated the molecular mechanism underlying GBM progression after radiotherapy by establishing a GBM orthotopic xenograft mouse model. Based on transcriptomic analysis, we found that the expression of BEX1 and BEX4 was upregulated in GBM cells surviving radiotherapy. We also found that upregulated expression of BEX1 and BEX4 was involved in the formation of the filamentous cytoskeleton and altered mechanotransduction, which resulted in the activation of the YAP/TAZ signaling pathway. BEX1- and BEX4-mediated YAP/TAZ activation enhanced the tumor formation, growth, and radioresistance of GBM cells. Additionally, latrunculin B inhibited GBM progression after radiotherapy by suppressing actin polymerization in an orthotopic xenograft mouse model. Taken together, we suggest the involvement of cytoskeleton formation in radiation-induced GBM progression and latrunculin B as a GBM radiosensitizer.

## 1. Introduction

Glioblastoma multiforme (GBM) is a highly advanced brain tumor derived from glial cells, including oligodendrocytes, astrocytes, and neural stem cells [1]. Surgical removal is widely performed as a treatment for GBM, followed by radiotherapy and chemotherapy to eliminate tumor cells in the marginal region of the tumor. However, the prevalence of high therapeutic resistance and recurrence of GBM prevents complete tumor control and results in short survival expectancy, with a median survival of about 15 months [2]. As the recurrence rate of GBM is nearly 100% and the cells show higher resistance to antitumor therapies, it is necessary to regulate the occurrence of the acquired therapeutic resistance of GBM [3]. In a previous study, transcriptomic profiles in orthotopically xenografted GBM cells surviving radiotherapy were analyzed, and a therapeutic strategy to target metabolic reprogramming was suggested [4]. However, there are still some aspects to be discovered to overcome GBM resistance.

The brain-expressed X-linked (*BEX*) family consists of five member genes (from 1 to 5) commonly located on the X chromosome. The molecular weight of BEXs is approximately 15 kDa, but little is known about their structural characteristics and molecular functions [5]. Since members of the BEX family are also p75 neurotrophin receptor-associated cell death executors (NADEs), most studies on BEXs have reported their involvement in the regulation of apoptosis and some signaling pathways in tumor cells [6,7]. Previous studies have suggested the oncogenic or tumor-suppressive roles of BEXs, with much controversy. Additionally, some studies have provided evidence of the involvement of BEXs in various biological events, such as the regulation of muscle regeneration, neuronal regeneration, development, and autophagy [8,9,10,11]. However, a large portion of the roles of BEXs remain elusive, and their radiation response has not been reported.

The arrangement of the cytoskeleton significantly affects tumor formation and growth. In particular, actin filaments, which are formed by the polymerization of globular actin (G-actin) to filamentous actin (F-actin), determine cell motility and activate signaling pathways, such as Ca^2+^, α-catenin/vinculin, and YAP/TAZ, for cellular states [12,13,14]. Among these, the involvement of YAP/TAZ signaling in mechanotransduction has recently garnered increasing attention. Yorkie (ortholog of YAP/TAZ) was first found in *Drosophila* as an effector of the Hippo signaling pathway, and its role in the maintenance of tissue homeostasis has been widely studied in mammalian cells. In particular, activated YAP/TAZ results in elevated tumorigenicity, proliferation, drug resistance, epithelial-to-mesenchymal transition (EMT), and metastasis in various types of tumor cells with poor prognosis in patients [15]. However, in glioma, only limited information about the roles of YAP/TAZ in invasion has been reported.

In this study, we established a GBM orthotopic xenograft mouse model to investigate the molecular events occurring in surviving GBM cells following radiotherapy. Based on the result of the transcriptomic analysis, we observed the upregulation of *BEX1* and *BEX4* expression in post-irradiation GBM cells and discovered downstream molecular events that account for GBM aggressiveness.

## 2. Results

### 2.1. Expression of BEX1 and BEX4 Was Elevated in Radioresistant GBM Cells

**Figure 1:**
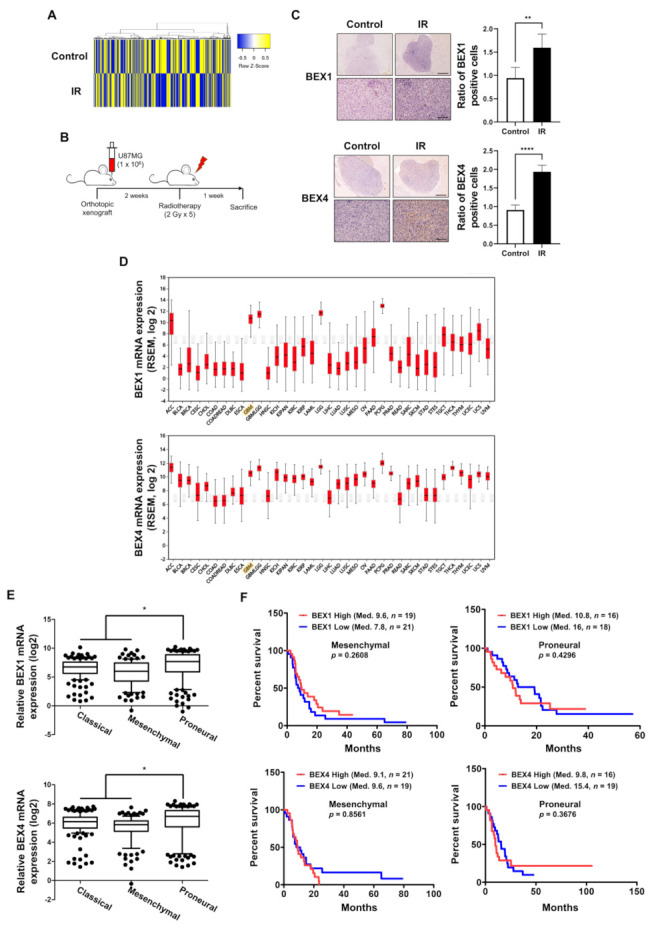
The expression levels of *BEX1* and *BEX4* were elevated in radioresistant GBM cells. (**A**) The heatmap of differentially expressed genes in the ‘neuronal development’ ontological group between control and irradiated GBM samples. (**B**) The schematic description of the generation of orthotopic xenograft GBM mouse model and the irradiation schedule. (**C**) Immunohistochemistry (anti-BEX1 and anti-BEX4) of coronal sections from mice bearing U87MG xenografts, control or treated with IR. BEX1 or BEX4 positive cells were quantified by ImageJ (*n* = 6). Scale bars, 500 μm (upper) or 100 μm (lower). (**D**) The differential expression of *BEX1* and *BEX4* according to the cancer types analyzed with the firebrowse database. (**E**) The differential mRNA expression of *BEX1* and *BEX4* according to the GBM subtypes. The numeric data were obtained from GlioVis. (**F**) The differential prognosis of patients with mesenchymal or proneural GBM according to the mRNA expression of *BEX1* and *BEX4*. The numeric data were obtained from GlioVis. Statistical analysis was performed with Student’s *t*-test for (**C**) and one-way ANOVA plus a Tukey’s multiple comparisons test for (**E**). * *p* < 0.05, ** *p* < 0.01, **** *p* < 0.0001.

GBM cells surviving radiotherapy often show enhanced radioresistance and progressive phenotypes, resulting in poor prognosis in GBM patients. To investigate the underlying biological events in these GBM cells, we analyzed the molecular changes in GBM cells following radiotherapy, based on the microarray results of a previous study to screen differentially expressed genes using radiotherapy [4]. The heatmap in Figure 1A shows significant differences in gene expression between the two samples, and we observed that the expression of *BEX1* and *BEX4* was increased by irradiation. To confirm the microarray data, we established an orthotopic GBM xenograft mouse model and verified the expression of *BEX1* and *BEX4* in the GBM cells surviving radiotherapy (Figure 1B). As shown in Figure 1C, the increased expression of these two genes was validated by IHC in GBM tissues from the mouse models. To confirm the involvement of these genes with GBM progression in patients, we utilized bioinformatics databases to assess the clinical significance of their expression. According to the firebrowse database, the expression levels of *BEX1* and *BEX4* were considerably higher in GBM than in other types of cancers, suggesting that *BEX1* and *BEX4* may play an important role in GBM progression (Figure 1D). Additionally, the analysis from GlioVis based on a recent glioma dataset, the Chinese Glioma Genome Atlas (CGGA), suggested that the expression of *BEX1* and *BEX4* was significantly different according to the subtypes (*p*-value ≤ 0.0001 or 0.0012, respectively, ANOVA) and was the highest in the proneural subtype (Figure 1E). Next, we analyzed the relationship between the expression of each gene and GBM patient survival. Although neither *BEX1* nor *BEX4* expression was significantly related to the survival of patients with GBM (data not shown, *n* = 220), a high expression of *BEX1* or *BEX4* in proneural GBM marginally reduced the median survival of patients (Figure 1F). However, the roles of *BEX1* and *BEX4* in survival in the mesenchymal subtype were opposite to those in the proneural subtype. In summary, the expression of *BEX1* and *BEX4* was elevated in radioresistant GBM cells and specifically involved in proneural-subtype GBMs according to the databases.

### 2.2. BEX1 and BEX4 Enhanced the Progression of GBM

**Figure 2:**
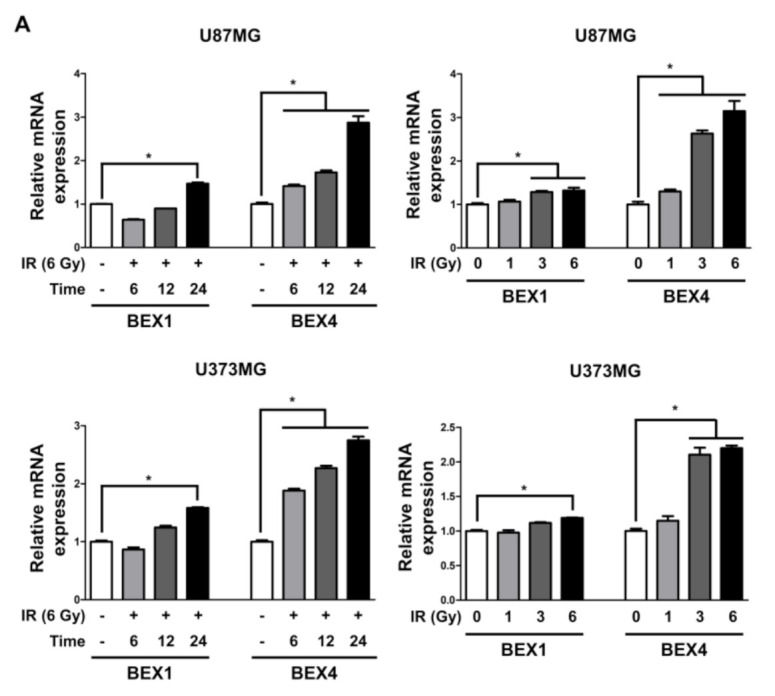

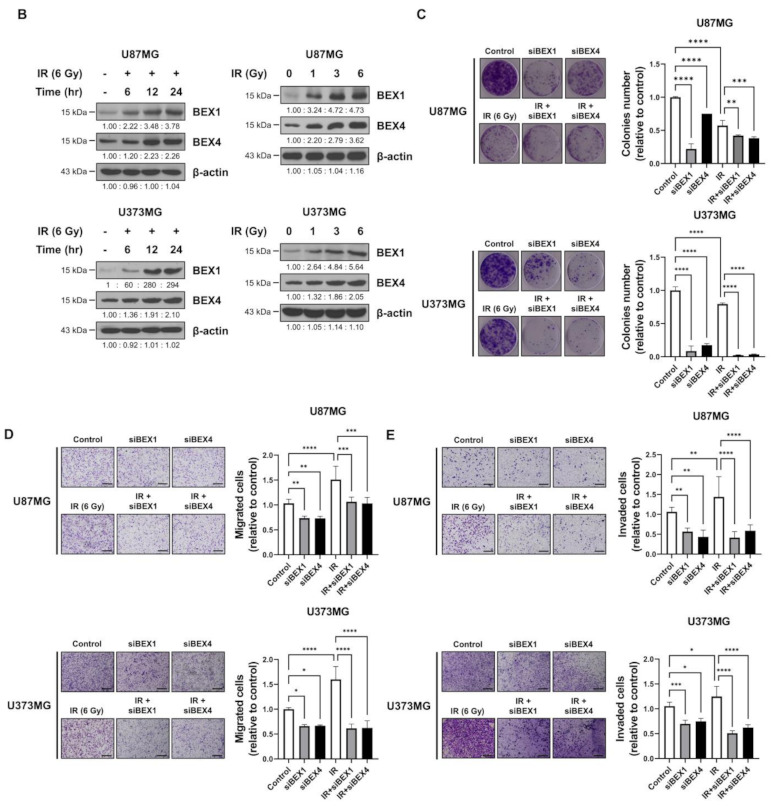
BEX1 and BEX4 increase migration and invasion of GBM. (**A**,**B**) The time- or dose-dependent alterations in the mRNA and protein expression levels of BEX1 and BEX4 after irradiation in U87MG and U373MG were assessed by qRT-PCR and Western blot analysis. (**C**) The proliferation of U87MG and U373MG cells after knockdown of *BEX1* or *BEX4* with/without irradiation was assessed by clonogenic assay, and the colonies were counted by openCFU software (*n* = 3). (**D**,**E**) The migration and invasion ability of U87MG and U373MG cells after knockdown of *BEX1* or *BEX4* with/without irradiation was assessed by Transwell migration or invasion assay and quantified by ImageJ (*n* = 3). Statistical analysis was performed with one-way ANOVA plus a Tukey’s multiple comparisons test. * *p* < 0.05, ** *p* < 0.01, *** *p* < 0.001, **** *p* < 0.0001.

Based on the elevated expression of *BEX1* and *BEX4* in radioresistant GBM, we investigated the involvement of the two genes in GBM progression enhanced following radiotherapy. In representative GBM cell lines, U87MG and U373MG, the delivery of radiation increased both the mRNA and protein expression of BEX1 and BEX4 in a time-dependent manner and 24 h after 6 Gy of IR was selected as the optimized condition for further experiments (Figure 2A,B and Supplementary Figure S1). This result suggested that radiation itself can cause an elevated expression of BEX1 and BEX4, as shown in GBM mouse models. Next, to validate the role of the expression of both genes in survival, proliferation, migration, and invasion, we conducted clonogenic, migration, and invasion assays upon the treatment of *BEX1* or *BEX4* siRNA with or without irradiation (Supplementary Figure S2). As shown in Figure 2C, knockdown of either *BEX1* or *BEX4* markedly reduced the survival of GBM cells, which was further reduced when combined with radiation. In the migration assay, radiation significantly increased the migration ability of GBM cells, and knockdown of *BEX1* or *BEX4* prevented their migration (Figure 2D). Furthermore, the differences caused by the knockdown were more significant when combined with irradiation. The results of the invasion assay showed that knockdown of either *BEX1* or *BEX4* markedly reduced the invasiveness of GBM cells both with and without irradiation (Figure 2E). These results validated the finding that radiation increases the expression of *BEX1* and *BEX4*, leading to increased survival, proliferation, and invasion of GBM cells, and suggested the importance of the suppression of the two genes to prevent the acquisition of aggressiveness of GBM.

### 2.3. BEX1 and BEX4 Modulated GBM Progression through the Regulation of Mechanotransduction

**Figure 3:**
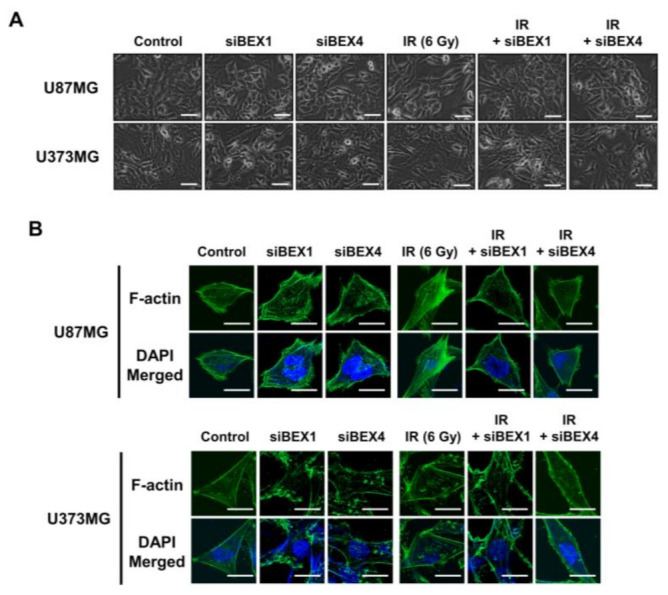

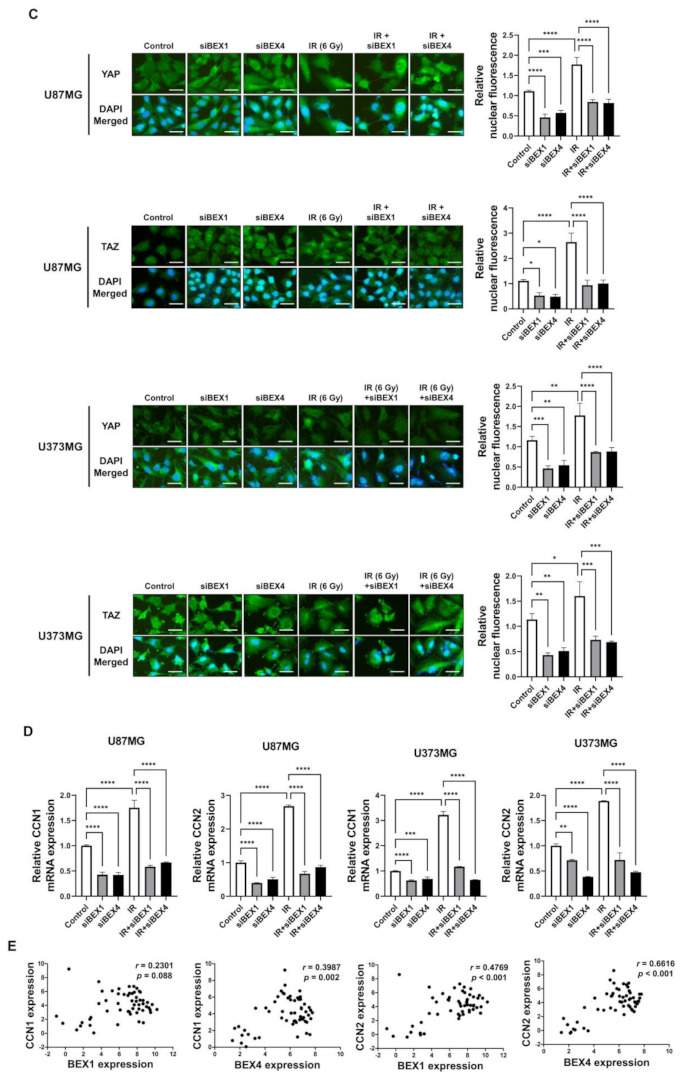
BEX1 and BEX4 modulate GBM progression through regulation of mechanotransduction. (**A**) The morphological changes in U87MG after knockdown of *BEX1* or *BEX4* with/without irradiation were photographed. (**B**) The formation of F-actin in U87MG after knockdown of *BEX1* or *BEX4* with/without irradiation was visualized by phalloidin staining assay. F-actin was stained green and nucleus was stained blue. (**C**) The alteration of subcellular localization of YAP or TAZ in U87MG after knockdown of *BEX1* or *BEX4* with/without irradiation was visualized by IF. YAP or TAZ was stained green and nucleus was stained blue. The nuclear localization of YAP or TAZ was quantified by ImageJ (*n* = 8). (**D**) The mRNA expression levels of *CCN1* and *CCN2* in U87MG after knockdown of *BEX1* or *BEX4* with/without irradiation were assessed by qRT-PCR. (**E**) Pearson’s correlation plots indicating gene expression levels of *BEX1*, *BEX4*, *CCN1*, and *CCN2* in proneural GBM patients from the CGGA database. Pearson’s correlation coefficient (r) and *p*-values are shown for each analysis. Statistical analysis was performed with one-way ANOVA plus a Tukey’s multiple comparisons test for (**C**,**D**). * *p* < 0.05, ** *p* < 0.01, *** *p* < 0.001, **** *p* < 0.0001.

BEX1 was shown to play a significant role in axon regeneration after neuronal damage, although the molecular mechanism is unknown. Since axon regeneration processes include the polymerization of microtubules and actin filaments, we hypothesized that alteration in cytoskeleton formation is mediated by BEX1 and BEX4 to accelerate cytoskeleton-mediated tumor progression. As shown in Figure 3A, irradiation induced GBM cells to elongate into a mesenchymal morphology with more branches, while knockdown of *BEX1* or *BEX4* prevented this and sustained a cobblestone-like morphology after irradiation. Between the two core cytoskeletal components, microtubules and actin filaments, previous studies emphasized the primary involvement of actin filaments in morphological changes and motility of cells [16]. Therefore, we specifically stained filamentous actin (F-actin) with phalloidin. As shown in Figure 3B, irradiation induced a more rigid and compact formation of F-actin, while knockdown of *BEX1* or *BEX4* reduced the formation of F-actin, which was more remarkable in irradiated GBM cells. Next, we investigated whether F-actin formation initiated by BEX1 or BEX4 can be relayed to downstream signaling transduction, so-called mechanotransduction. Among the cellular mechanical transcription factors, YAP/TAZ has been found to be crucial in tumor progression. First, based on the fact that the activity of YAP/TAZ is determined by nuclear localization, we assessed the intracellular localization of the proteins. Irradiation increased the nuclear accumulation of YAP and TAZ, which was suppressed by the knockdown of *BEX1* and *BEX4* (Figure 3C). To validate the activity of YAP and TAZ, the expression of *CCN1* and *CCN2*, representative transcriptional target genes of YAP/TAZ, was measured. As shown in Figure 3D, radiation significantly increased the expression of both genes, and knockdown of *BEX1* or *BEX4* prevented these effects. The remarkable association among *BEX1*, *BEX4*, *CCN1*, and *CCN2* was validated using the CGGA database. As shown in Figure 3E, proneural-subtype GBM showed significant correlations in the expression of each pair of genes, supporting the BEX1- and BEX4-induced transcriptional activity of YAP/TAZ. Therefore, radiation-induced BEX1 and BEX4 activated mechanotransduction through the formation of F-actin and enhanced the transcriptional activation of YAP/TAZ through nuclear localization, which was confirmed using information from the clinical GBM database.

### 2.4. Regulation of F-Actin Polymerization Reduced Radiation-Induced GBM Progression

**Figure 4:**
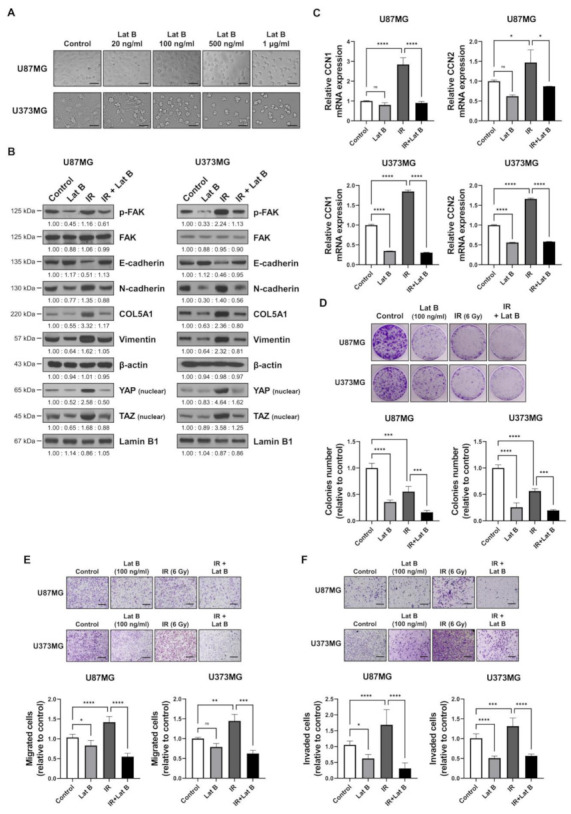

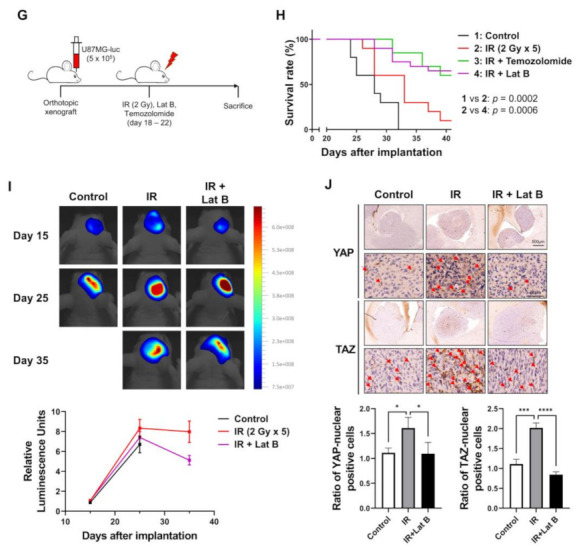
Regulation of F-actin polymerization reduces radiation-induced GBM progression. (**A**) The dose-dependent morphological changes in U87MG after the treatment of Lat B were photographed. (**B**) The expression of proteins involved in migration in U87MG and U373MG was assessed by Western blot analysis upon treatment of Lat B (100 ng/mL), IR (6 Gy), or Lat B with IR. (**C**) The mRNA expression levels of *CCN1* and *CCN2* in U87MG after the treatment of Lat B were assessed by qRT-PCR. * *p* < 0.05, ** *p* < 0.01, *** *p* < 0.001. (**D**) The proliferation of U87MG cells after the treatment of Lat B was assessed using a clonogenic assay, and the colonies were counted by openCFU software (*n* = 3). (**E**,**F**) The migration and invasion ability of U87MG cells after the treatment of Lat B was assessed by Transwell migration or invasion assay and quantified by ImageJ (*n* = 3). (**G**) A schematic diagram of control, IR (2 Gy × 5), IR with temozolomide (20 mg/kg, intraperitoneal), and IR with Lat B (25 μg/kg, intracranial) treatment in mice bearing U87MG-luciferase xenografts (*n* = 20 mice per group). (**H**) Survival analysis by Kaplan–Meier curves and log-rank (Mantel–Cox) test of mice bearing U87MG-luciferase xenografts control, treated with IR, IR with temozolomide, or IR with Lat B. (**I**) In vivo bioluminescent images of orthotopic xenografts derived from U87MG-luciferase in control mice, treated with IR, or IR with Lat B. The images were quantified by the ROI measurement tool from VISQUE Invivo Smart LF software. (**J**) Immunohistochemistry (anti-YAP and anti-TAZ) of coronal sections from mice bearing U87MG-luciferase xenografts, control, treated with IR, or IR with Lat B. The ratio of YAP- or TAZ-nuclear positive cells was quantified by ImageJ (*n* = 6). Arrows indicate nuclear accumulation of YAP or TAZ. Statistical analysis was performed with one-way ANOVA plus a Tukey’s multiple comparisons test. * *p* < 0.05, ** *p* < 0.01, *** *p* < 0.001, **** *p* < 0.0001.

**Figure 5:**
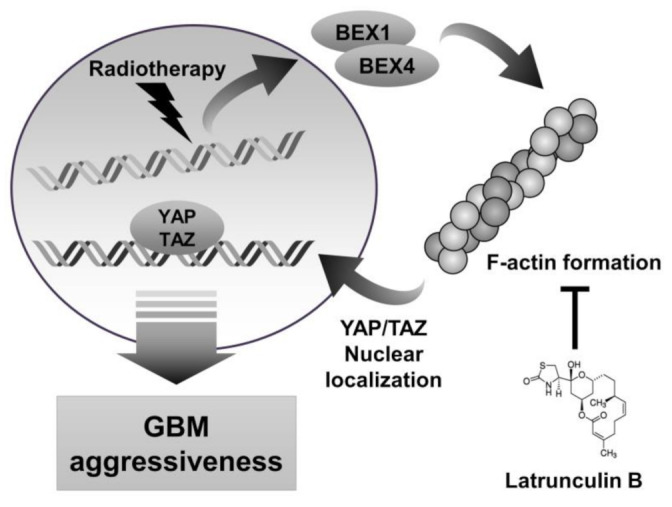
Schematic diagram depicting that inhibition of actin polymerization by latrunculin B reduces GBM aggressiveness. IR-induced BEX1 and BEX4 lead to actin polymerization, which localizes YAP/TAZ to nuclear. YAP/TAZ nuclear translocation induces GBM aggressiveness by activating the downstream target genes.

We observed that the radiation-induced expression of BEX1 and BEX4 led to F-actin polymerization and the acquisition of migratory and invasive capacities through the activation of mechanotransduction. We hypothesized that the regulation of F-actin polymerization prevents radiation-induced GBM progression and increases therapeutic efficacy. Latrunculin (Lat) A and B, natural products from marine sponges, reportedly inhibit F-actin polymerization, and Lat A exerted stronger biological effects than Lat B [17]. Nevertheless, we adopted Lat B as an alternative regulator of F-actin polymerization in GBM cells because intracranial injection of Lat A was reported to induce seizures [18]. To determine the concentration of Lat B for use as an adjuvant radiosensitizer, we screened the biological effects of Lat B treatment. As shown in Figure 4A, we observed morphological changes at a concentration of 100 ng/mL. Next, we confirmed that the expression of EMT markers altered by irradiation was recovered by Lat B treatment to acquire epithelial phenotypes in GBM cells (Figure 4B and Supplementary Figure S3). Additionally, aberrant F-actin polymerization led to a decrease in the transcriptional activity of YAP and TAZ. Lat B treatment significantly suppressed the expression of *CCN1* and *CCN2*, and the effects of radiation on the expression were diminished (Figure 4C). In the clonogenic assay, Lat B treatment reduced colony growth, while the number of colonies did not change (Figure 4D). In combination with irradiation, Lat B significantly reduced both the number and size of colonies. In the migration assay, migration was markedly reduced by Lat B treatment, and the difference was greater in irradiated cells (Figure 4E). The results of the invasion assay also indicated that Lat B treatment markedly reduced the invasive ability of GBM cells (Figure 4F). To examine the effects of Lat B in vivo, we orthotopically implanted U87MG-luciferase-expressing cells (U87MG-luc) in BALB/c nude mice. The mice were treated with IR alone or IR in combination with Lat B or TMZ 18 days after the orthotopic xenograft (Figure 4G). Consequently, Lat B significantly extended the average survival of tumor-bearing mice and was as effective as TMZ, a standard treatment for GBM, in combination with IR (Figure 4H). Furthermore, in vivo bioluminescent imaging showed that the IR/Lat B combination significantly inhibited tumor growth (Figure 4I). To investigate the expression and localization of YAP/TAZ in combination with IR/Lat B, we performed a histological analysis of the brain tissue. As shown in Figure 4J, YAP/TAZ significantly increased and accumulated in the nucleus following IR, and their levels were rescued by IR/Lat B combination therapy. In summary, Lat B treatment reversed the infiltrative ability of GBM cells and improved the survival of GBM-bearing mice after irradiation by suppressing F-actin cytoskeleton formation (Figure 5).

## 3. Discussion

Although a variety of biological roles of BEXs have been previously reported, we first suggested BEX1- and BEX4-mediated cytoskeleton formation and migration in GBM. BEX1 is the most widely investigated member of the BEX family and is involved in myogenesis, apoptosis, the activation of NF-kB, neuronal homeostasis, and early development, and BEX4 reportedly regulates apoptosis and aneuploidy [9,19,20,21,22]. Among these, the involvement of BEX1 in myogenesis and that of BEX4 in aneuploidy may suggest a biological relationship with actin filaments and microtubules, respectively. In addition, BEX1 and BEX4 may regulate the GBM migratory ability in a synergistic manner because cellular migration is mediated by filopodia formation consisting of actin filaments and microtubules [23]. Furthermore, BEX1- and BEX4-mediated F-actin polymerization was related to the activation of downstream signaling, providing more direct evidence of their biological roles in cytoskeleton regulation. However, biochemical investigations of their roles have not been performed, and detailed basic research is needed for a better understanding.

Each member of the BEX family has distinct biological effects, even though their genomic location and sequences are similar to each other. A large number of studies have reported that BEX2 increases migratory activity and inhibits the apoptosis of tumor cells by activating JNK and NF-κB signaling [24,25,26]. BEX3 has the most distinctive functions, directly interacting with p75NTR to induce apoptosis [27,28]. The molecular functions of BEX5 have not been well studied. Despite their contributions to tumor progression, the expression of three genes was not shown to be differentially expressed with radiotherapy (1.049-fold in *BEX2*, 1.057-fold in *BEX3*, and 1.064-fold in *BEX5*) in the microarray results. Although we could not rule out their roles in GBM progression, they are not supposed to be related to acquired GBM aggressiveness after radiotherapy. Conversely, a study provided evidence that the correlated expression of BEX1 and BEX4 is associated with lung adenocarcinoma prognosis [29]. Therefore, the differential transcriptional regulation of each member of the BEX family may determine the involvement and fate of tumor cells.

In this study, we suggest that Lat B inhibits radioresistance acquisition and GBM progression by suppressing F-actin formation. The significant involvement of cytoskeleton organization in the migration and invasion of tumor cells has been widely studied. Actin filaments, along with microtubules, function as the core cytoskeleton and regulate the motility of tumor cells through the formation of the principal structure of filopodia [30]. Although the inhibition of cytoskeleton formation could be life-threatening, the use of inhibitors for F-actin formation, such as jasplakinolide, cytochalasin D, and Lat A, has previously been identified as a promising strategy to suppress tumor malignancy [31,32,33]. Given these observations, it has remarkable potential and further studies are needed to evaluate the safety of Lat B.

Many studies have reported the significant role of YAP/TAZ in tumor progression. As a major mechanotransductive signaling pathway, the transcriptional activity of YAP/TAZ determines the mobile characteristics of tumor cells. Although a few studies have been conducted on GBM cells, some studies have shown that YAP/TAZ signaling pathways worsen the prognosis of GBM patients [34,35]. In the same context, some studies provided molecular evidence for the significant involvement of proteins such as transcriptional co-activators, kinases, and membrane proteins in the promotion of GBM progression via the activation of the YAP/TAZ signaling pathway [36,37]. In addition, in the studies covering the GBM subtypes where YAP/TAZ signaling is majorly involved, the proneural subtype was mainly affected by the YAP/TAZ signaling pathway, while the significance of classical and mesenchymal subtypes was controversial [37]. Considering the distinctive involvement of BEX1 and BEX4 expression in the survival of proneural GBM patients, it suggests the potential clinical relevance of our findings.

## 4. Materials and Methods

### 4.1. Chemicals, Antibodies, and Reagents

Latrunculin B was purchased from Cayman Chemicals (Ann Arbor, MI, USA) and Phalloidin-iFluor 488 reagent (Cytopainter) was purchased from Abcam (Cambridge, MA, USA). Antibodies specific for BEX1 and BEX4 were purchased from Abcam and those specific for p-FAK, E-cadherin, N-cadherin, COL5A1, vimentin, YAP, TAZ, and β-actin were purchased from Santa Cruz Biotechnology (Santa Cruz, CA, USA). Dulbecco’s modified Eagle’s medium (DMEM), fetal bovine serum (FBS), penicillin, streptomycin, and TRIzol were obtained from Thermo Fisher Scientific (Waltham, MA, USA). siRNAs specific for mRNA of *BEX1*, *BEX4*, *YAP*, and *TAZ* were purchased from Bioneer (Daejeon, Republic of Korea) (Table S2).

### 4.2. Cell Culture and Treatment

The human glioblastoma cell lines U87MG and U373MG were cultured as described in a previous study [38]. Briefly, the cells were cultured in MEM medium supplemented with 10% FBS and 1% penicillin/streptomycin and kept in an incubator at 37 °C with 5% CO_2_ and humidity. To assess the effects of treatment, the cells were incubated in serum-free media 24 h before treatment. The cells with confluence of approximately 70% were used for the experiments. X-rays were delivered by an X-ray generator M-150WE (Softex, Tokyo, Japan).

### 4.3. Animal Care Protocol and Orthotopic Xenograft Mouse Model

Following a previous study, six-week-old male BALB/c athymic nude mice (Orient Bio, Seongnam, Korea) were used to generate a xenograft mouse model [4]. The mice were housed individually or in groups of up to five in sterile cages and maintained in animal care facilities in a temperature-regulated room (23 ± 1 °C) with a 12 h light–dark cycle. All animals were fed water and standard mouse chow ad libitum. U87MG-luciferase-expressing cells were harvested through trypsinization and suspended at a density of 1 × 10^5^ cells/μL in serum-free media. Next, 5 × 10^5^ cells were injected at stereotactic coordinates of bregma, −1 mm anteroposterior, and +2 mm mediolateral using stereotaxic injection frame. Eighteen days after the injection date, 5 μL of Lat B dissolved in ethanol was intracranially injected using the stereotaxic injection frame at the infusion rate of 0.5 μL/min and 5 min of retention time after infusion. After the cranial injection, the mouse brains were irradiated with 2 Gy/d for five days at a dose rate of 600 MU/min using a TrueBeam STx (Varian Medical Systems, Palo Alto, CA, USA). Xenograft growth was monitored by bioluminescent imaging using a VISQUE Invivo Smart LF (Vieworks, Anyang, Republic of Korea). Mice were euthanized when they became moribund to the extent that they were found to be immobile and unresponsive.

### 4.4. Ethics Statement

The animal protocol used in this study was approved by the Pusan National University Institutional Animal Care and Use Committee (PNU-IACUC) for ethical procedures and scientific care (Approval Number PNU-2020-2809) on 2 December 2020.

### 4.5. Database Analysis

The firebrowse database was used for analyzing the differential expression of BEX1 and BEX4 in various cancer types (accessed on 23 August 2021). The GlioVis tool was utilized for analyzing the expression of the genes according to GBM subtype and prognosis of the patients (accessed on 23 August 2021) [39]. The numeric data were obtained from GlioVis, and graphs were drawn using Prism 5 software (GraphPad Software, San Diego, CA, USA).

### 4.6. Immunofluorescence (IF) and Phalloidin Staining Assay

For IF or phalloidin staining, cells were fixed with 4% paraformaldehyde in phosphate-buffered saline (PBS) for 20 min, permeabilized in ice-cold acetone for 10 min, washed three times with PBS, and blocked in blocking buffer (0.1% BSA in PBS) for 30 min. Cells were stained with primary antibody against YAP and TAZ or phalloidin staining solution overnight at 4 °C and washed three times with PBS. After incubation with DyLight 488-conjugated secondary antibodies (Thermo Scientific, Cleveland, OH, USA) and counterstaining with 4,6-diamidino-2-phenylindole, slides were mounted with VECTASHIELD Hard-Set Mounting Medium (Vector Laboratories, Burlingame, CA, USA). Fluorescent images were visualized using a Leica DMi 8 fluorescence microscope (Leica, Wetzlar, Germany). The nuclear localization of YAP and TAZ was quantified using the ImageJ colocalization plugin and validated by statistical analyses.

### 4.7. Immunohistochemistry (IHC)

IHC was conducted as described previously [40]. Brain samples were embedded in paraffin, and tissue sections were prepared using HistoCore AutoCut (Leica, Deerfield, IL, USA). Next, the sections were treated with 3% hydrogen peroxide/methanol and with 0.25% pepsin to retrieve antigens. The samples were incubated in blocking solution (Dako, Carpinteria, CA, USA), and were incubated at 4 °C overnight with the primary antibodies diluted in the antibody diluent (Dako). Then, the sections were washed with Tris-buffered saline with 0.1% Tween 20 and incubated with a HRP conjugated secondary antibody (Dako). A 3,3′-diaminobenzidine substrate chromogen system (Dako) was used to detect antibody binding. Stained sections were visualized with an Olympus IX71 inverted microscope (Olympus Optical, Tokyo, Japan). The IHC images were quantified by transforming images into positive/negative pixels using ImageJ and validated by statistical analyses.

### 4.8. Clonogenic Assay

A clonogenic assay to assess cell viability and proliferation after treatment was performed as described in a previous study [41]. Cells were seeded at a density of 500 cells in 35-mm dishes, and 24 h later, they were treated with siRNAs for BEX1, BEX4, or Lat B. After 24 h of treatment, the cells were grown at 37 °C in a 5% CO_2_/95% air atmosphere for 14 d. Next, the cells were fixed with 10% methanol/10% acetic acid, stained with 1% crystal violet, and scanned for data acquisition.

### 4.9. Total RNA Isolation and qRT-PCR

For mRNA expression assessment, qRT-PCR was performed as described previously [4]. Briefly, RNA was isolated with TRIzol, following the manufacturer’s instructions, and real-time qRT-PCR was conducted by using an Applied Biosystems StepOne Real-Time PCR System (Applied Biosystems, Foster City, CA, USA). It was performed for 40 cycles at 95 °C for 15 s and 60 °C for 1 min, followed by thermal denaturation. The primer sequences used are listed in Supplementary Table S1.

### 4.10. Western Blot Analysis

Protein expression was measured as described previously [42]. Briefly, whole cell lysates (WCL) were prepared using radioimmunoprecipitation assay (RIPA) lysis buffer (50 mM Tris, pH 7.4, 150 mM NaCl, 1% Triton X-100, 25 mM NaF, 1 mM dithiothreitol (DTT), and 20 mM EGTA supplemented with protease inhibitors), and the protein concentrations were measured using a Bio-Rad protein assay kit (Bio-Rad Laboratories, Hercules, CA, USA). Protein samples were subjected to SDS-PAGE, transferred to a nitrocellulose membrane, and blocked with 5% bovine serum albumin in TBST (10 mM Tris, 100 mM NaCl, and 0.1% Tween 20). Next, membranes were probed with primary antibodies and peroxidase-conjugated secondary antibodies (Santa Cruz Biotechnology, Santa Cruz, CA, USA). The membranes were analyzed using an ECL detection system (Roche Applied Science, Indianapolis, IN, USA) with iBright chemi-doc fl000 from Thermo Fisher Scientific.

### 4.11. Transwell Cell Migration/Invasion Assay

Effects of knockdown of BEX1, BEX4, YAP, or TAZ and treatment of Lat B on cell migration/invasion ability were investigated by Transwell cell migration/invasion assay, as previously described [43]. Cells (5 × 10^4^ in serum-free MEM medium) were seeded into the upper chambers of a 24-well Transwell chamber (Corning, Corning, NY, USA) fitted with a 5-μm pore size insert and treated with siRNA, Lat B, and/or radiation, for 24 h. Next, the lower chamber was changed into MEM medium containing 2% FBS. After 12 or 24 h, the upper membrane surface was wiped with a cotton swab to remove cells that had not migrated to the lower side of the membrane. The upper chambers were fixed with 70% EtOH, stained with 0.05% crystal violet, and photographed using an AXIO microscope (Carl Zeiss, Oberkochen, Germany). The images of migration and invasion assays were quantified by counting the number of cells on the underside of the membrane using ImageJ and validated by statistical analyses.

### 4.12. Statistical Analysis

All numerical data are presented as the mean ± standard error from at least three independent experiments. For quantification, the data were analyzed using *t*-tests or ANOVA. Prism 5 software was used for all statistical analyses. Statistical significance was set at *p* < 0.05.

## 5. Conclusions

We found that BEX1 and BEX4 induced F-actin polymerization and the activation of the YAP/TAZ signaling pathway, which led to GBM aggressiveness. As an inhibitor of the acquired radioresistance and progression of GBM cells, we suggest that Lat B has experimental significance. The findings that BEX1 and BEX4 mediate cytoskeleton formation in GBM cells after radiotherapy and treatment with Lat B, a pharmacological candidate, can overcome acquired GBM aggressiveness may lead to a therapeutic strategy.

## Data Availability

The accession number for the cDNA microarray analysis data reported in this paper is GEO: GSE117126.

## Supplementary Materials

The following are available online at https://www.mdpi.com/article/10.3390/ijms22189845/s1, Figure S1: The time- or dose-dependent alterations in the mRNA and protein expression levels of BEX1 and BEX4 after irradiation in U87MG and U373MG, Figure S2: Protein levels of BEX1 and BEX4 upon treatment of their corresponding siRNAs in U87MG and U373MG, Figure S3: The expression of proteins involved in migration in U87MG and U373MG, Table S1: Primers used for qRT-PCR to assess gene expression alterations, Table S2: Sequence of BEX1 and BEX4 siRNAs.

## References

[1] Zong H., Verhaak R.G., Canoll P. (2012). The cellular origin for malignant glioma and prospects for clinical advancements. Expert Rev. Mol. Diagn..

[2] Delgado-Martín B., Medina M. (2020). Advances in the Knowledge of the Molecular Biology of Glioblastoma and Its Impact in Patient Diagnosis, Stratification, and Treatment. Adv. Sci. (Weinh).

[3] Filley A.C., Henriquez M., Dey M. (2017). Recurrent glioma clinical trial, CheckMate-143: The game is not over yet. Oncotarget.

[4] Son B., Lee S., Kim H., Kang H., Jeon J., Jo S., Seong K.M., Lee S.J., Youn H., Youn B. (2020). Decreased FBP1 expression rewires metabolic processes affecting aggressiveness of glioblastoma. Oncogene.

[5] Fernandez E.M., Díaz-Ceso M.D., Vilar M. (2015). Brain expressed and X-linked (Bex) proteins are intrinsically disordered proteins (IDPs) and form new signaling hubs. PLoS ONE.

[6] Mukai J., Suvant P., Sato T.A. (2003). Nerve growth factor-dependent regulation of NADE-induced apoptosis. Vitam Horm.

[7] Kazi J.U., Kabir N.N., Rönnstrand L. (2015). Brain-Expressed X-linked (BEX) proteins in human cancers. Biochim. Biophys. Acta.

[8] Khazaei M.R., Halfter H., Karimzadeh F., Koo J.H., Margolis F.L., Young P. (2010). Bex1 is involved in the regeneration of axons after injury. J. Neurochem..

[9] Williams J.W., Hawes S.M., Patel B., Latham K.E. (2002). Trophectoderm-specific expression of the X-linked Bex1/Rex3 gene in preimplantation stage mouse embryos. Mol. Reprod. Dev..

[10] Fang X., Yoon J.G., Li L., Yu W., Shao J., Hua D., Zheng S., Hood L., Goodlett D.R., Foltz G. (2011). The SOX2 response program in glioblastoma multiforme: An integrated ChIP-seq, expression microarray, and microRNA analysis. BMC Genom..

[11] Colombo E., Romaggi S., Medico E., Menon R., Mora M., Falcone C., Lochmüller H., Confalonieri P., Mantegazza R., Morandi L. (2011). Human neurotrophin receptor p75NTR defines differentiation-oriented skeletal muscle precursor cells: Implications for muscle regeneration. J. Neuropathol. Exp. Neurol..

[12] Lange K., Gartzke J. (2006). F-actin-based Ca signaling-a critical comparison with the current concept of Ca signaling. J. Cell Physiol..

[13] Huveneers S., de Rooij J. (2013). Mechanosensitive systems at the cadherin-F-actin interface. J. Cell Sci..

[14] Park J.A., Kwon Y.G. (2018). Hippo-YAP/TAZ signaling in angiogenesis. BMB Rep..

[15] Park J., Shim J.K., Kang J.H., Choi J., Chang J.H., Kim S.Y., Kang S.G. (2018). Regulation of bioenergetics through dual inhibition of aldehyde dehydrogenase and mitochondrial complex I suppresses glioblastoma tumorspheres. Neuro-Oncology.

[16] Svitkina T. (2018). The Actin Cytoskeleton and Actin-Based Motility. Cold Spring Harb. Perspect. Biol..

[17] Helal M.A., Khalifa S., Ahmed S. (2013). Differential binding of latrunculins to G-actin: A molecular dynamics study. J. Chem. Inf. Model.

[18] Sierra-Paredes G., Loureiro A.I., Wright L.C., Sierra-Marcuño G., Soares-da-Silva P. (2014). Effects of eslicarbazepine acetate on acute and chronic latrunculin A-induced seizures and extracellular amino acid levels in the mouse hippocampus. BMC Neurosci..

[19] Jiang C., Wang J.H., Yue F., Kuang S. (2016). The brain expressed x-linked gene 1 (Bex1) regulates myoblast fusion. Dev. Biol..

[20] He M., Wang Y., Shen J., Duan C., Lu X., Li J. (2018). Bex1 attenuates neuronal apoptosis in rat intracerebral hemorrhage model. Pathol. Res. Pract..

[21] Koo J.H., Saraswati M., Margolis F.L. (2005). Immunolocalization of Bex protein in the mouse brain and olfactory system. J. Comp. Neurol..

[22] Lee J.K., Ha G.H., Kim H.S., Lee C.W. (2018). Oncogenic potential of BEX4 is conferred by Polo-like kinase 1-mediated phosphorylation. Exp. Mol. Med..

[23] Dogterom M., Koenderink G.H. (2019). Actin-microtubule crosstalk in cell biology. Nat. Rev. Mol. Cell Biol..

[24] Hu Y., Xiao Q., Chen H., He J., Tan Y., Liu Y., Wang Z., Yang Q., Shen X., Huang Y. (2017). BEX2 promotes tumor proliferation in colorectal cancer. Int. J. Biol. Sci..

[25] Zhou X., Xu X., Meng Q., Hu J., Zhi T., Shi Q., Yu R. (2013). Bex2 is critical for migration and invasion in malignant glioma cells. J. Mol. Neurosci..

[26] Hu Z., Wang Y., Huang F., Chen R., Li C., Wang F., Goto J., Kwiatkowski D.J., Wdzieczak-Bakala J., Tu P. (2015). Brain-expressed X-linked 2 Is Pivotal for Hyperactive Mechanistic Target of Rapamycin (mTOR)-mediated Tumorigenesis. J. Biol. Chem..

[27] Yoon K., Jang H.D., Lee S.Y. (2004). Direct interaction of Smac with NADE promotes TRAIL-induced apoptosis. Biochem. Biophys. Res. Commun..

[28] Mukai J., Hachiya T., Shoji-Hoshino S., Kimura M.T., Nadano D., Suvanto P., Hanaoka T., Li Y., Irie S., Greene L.A. (2000). NADE, a p75NTR-associated cell death executor, is involved in signal transduction mediated by the common neurotrophin receptor p75NTR. J. Biol. Chem..

[29] Zhang Z.H., Luan Z.Y., Han F., Chen H.Q., Liu W.B., Liu J.Y., Cao J. (2019). Diagnostic and prognostic value of the BEX family in lung adenocarcinoma. Oncol. Lett..

[30] Jacquemet G., Hamidi H., Ivaska J. (2015). Filopodia in cell adhesion, 3D migration and cancer cell invasion. Curr. Opin. Cell Biol..

[31] Eisemann T., Costa B., Strelau J., Mittelbronn M., Angel P., Peterziel H. (2018). An advanced glioma cell invasion assay based on organotypic brain slice cultures. BMC Cancer.

[32] Keurhorst D., Liashkovich I., Frontzek F., Nitzlaff S., Hofschröer V., Dreier R., Stock C. (2019). MMP3 activity rather than cortical stiffness determines NHE1-dependent invasiveness of melanoma cells. Cancer Cell Int..

[33] Sayed K.A., Khanfar M.A., Shallal H.M., Muralidharan A., Awate B., Youssef D.T., Liu Y., Zhou Y.D., Nagle D.G., Shah G. (2008). Latrunculin A and its C-17-O-carbamates inhibit prostate tumor cell invasion and HIF-1 activation in breast tumor cells. J. Nat. Prod..

[34] Liu Z., Yee P.P., Wei Y., Liu Z., Kawasawa Y.I., Li W. (2019). Differential YAP expression in glioma cells induces cell competition and promotes tumorigenesis. J. Cell Sci..

[35] Liu M., Lin Y., Zhang X.C., Tan Y.H., Yao Y.L., Tan J., Zhang X., Cui Y.H., Liu X., Wang Y. (2017). Phosphorylated mTOR and YAP serve as prognostic markers and therapeutic targets in gliomas. Lab. Investig..

[36] Yu O.M., Benitez J.A., Plouffe S.W., Ryback D., Klein A., Smith J., Greenbaum J., Delatte B., Rao A., Guan K.L. (2018). YAP and MRTF-A, transcriptional co-activators of RhoA-mediated gene expression, are critical for glioblastoma tumorigenicity. Oncogene.

[37] Cosset É., Ilmjärv S., Dutoit V., Elliott K., von Schalscha T., Camargo M.F., Reiss A., Moroishi T., Seguin L., Gomez G. (2017). Glut3 Addiction Is a Druggable Vulnerability for a Molecularly Defined Subpopulation of Glioblastoma. Cancer Cell.

[38] Son B., Jeon J., Lee S., Kim H., Kang H., Youn H., Jo S., Youn B. (2019). Radiotherapy in combination with hyperthermia suppresses lung cancer progression via increased NR4A3 and KLF11 expression. Int. J. Radiat. Biol..

[39] Bowman R.L., Wang Q., Carro A., Verhaak R.G., Squatrito M. (2017). GlioVis data portal for visualization and analysis of brain tumor expression datasets. Neuro-Oncology.

[40] Son B., Kwon T., Lee S., Han I., Kim W., Youn H., Youn B. (2017). CYP2E1 regulates the development of radiation-induced pulmonary fibrosis via ER stress- and ROS-dependent mechanisms. Am. J. Physiol. Lung. Cell Mol. Physiol..

[41] Kim W., Youn H., Kang C., Youn B. (2015). Inflammation-induced radioresistance is mediated by ROS-dependent inactivation of protein phosphatase 1 in non-small cell lung cancer cells. Apoptosis.

[42] Son B., Lee S., Kim H., Kang H., Kim J., Youn H., Nam S.Y., Youn B. (2019). Low dose radiation attenuates inflammation and promotes wound healing in a mouse burn model. J. Dermatol. Sci..

[43] Yang H.J., Youn H., Seong K.M., Yun Y.J., Kim W., Kim Y.H., Lee J.Y., Kim C.S., Jin Y.W., Youn B. (2011). Psoralidin, a dual inhibitor of COX-2 and 5-LOX, regulates ionizing radiation (IR)-induced pulmonary inflammation. Biochem. Pharmacol..

