# Supplementary material for: BEX1 and BEX4 Induce GBM Progression through Regulation of Actin Polymerization and Activation of YAP/TAZ Signaling
Source: Int J Mol Sci. 2021 Sep 11;22(18):9845. doi: 10.3390/ijms22189845 (PMC8471324; doi:10.3390/ijms22189845)
Supplement: Supplementary file 1 [file ijms-22-09845-s001.zip › ijms-1342205-supplementary materials.pdf]

Supplemental material for

BEX1 and BEX4 induce GBM progression through regulation of actin polymerization and activation of YAP/TAZ signaling

Sungmin Lee, Hyunkoo Kang, Eunguk Shin, Jaewan Jeon, HyeSook Youn, and BuHyun Youn

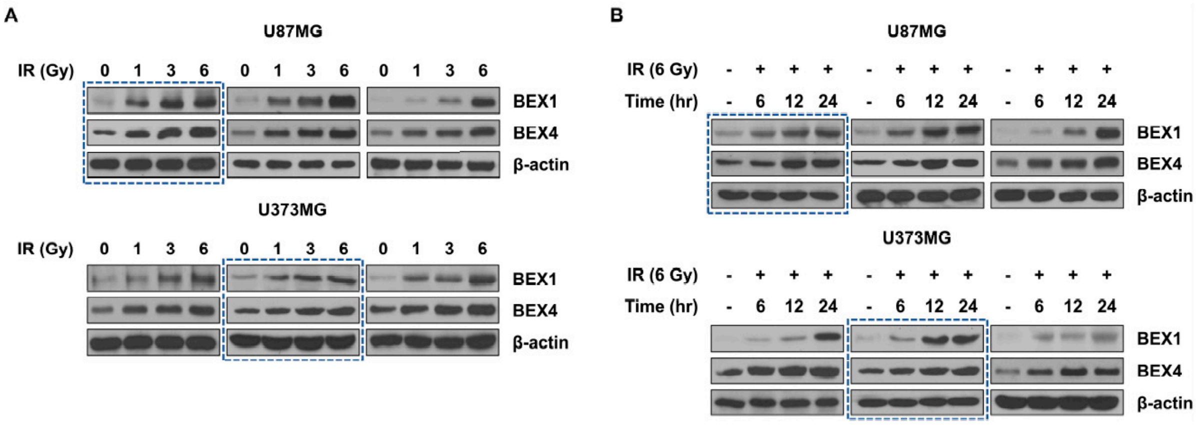

Supplemental Figure S1. (related to Figure 2B). (**A**) Dose-dependent alterations in the protein expression levels of BEX1 and BEX4 after irradiation in U87MG and U373MG were assessed by western blot analysis with biological triplicates. (**B**) Time-dependent alterations in the protein expression levels of BEX1 and BEX4 after irradiation in U87MG and U373MG were assessed by western blot analysis with biological triplicates. The data marked with a dotted line indicates the representative data in Figure 2B.

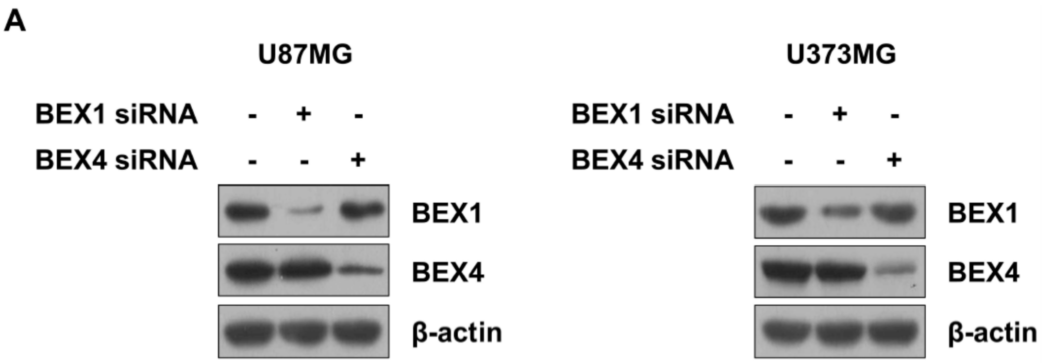

Supplemental Figure S2. (**A**) Protein levels of BEX1 and BEX4 were analyzed by Western blot upon treatment of their corresponding siRNAs in U87MG and U373MG cells.

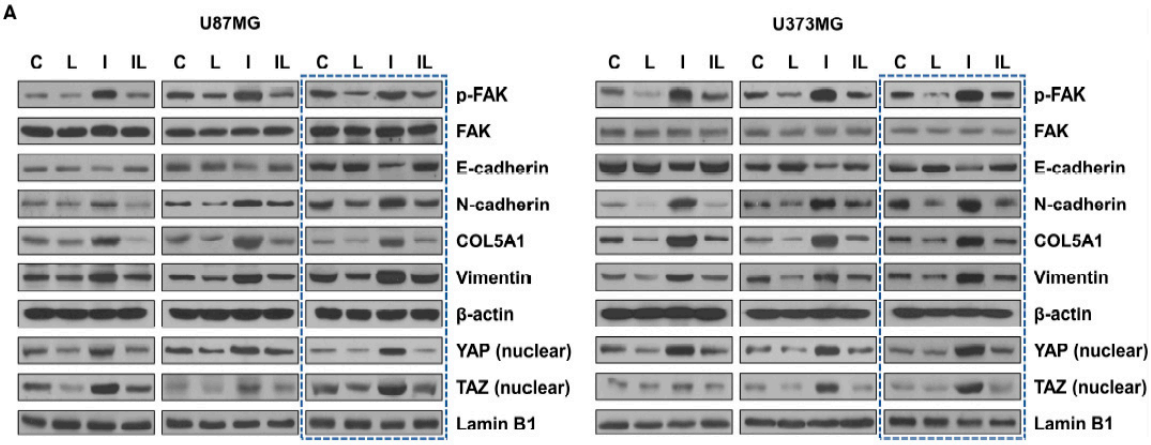

Supplemental Figure S3. (related to Figure 4B) (**A**) The expression of proteins involved in migration in U87MG and U373MG was assessed by western blot analysis with biological triplicates upon treatment of Lat B (100 ng/ml), IR (6Gy), or Lat B with IR. The data marked with a dotted line indicates the representative data in Figure 4B. C, Control; L, Lat B (100 ng/ml); I, IR (6 Gy); IL, IR with Lat B.

Supplemental Table S1. Primers used for qRT-PCR to assess gene expression alterations.

Gene name	Forward primer	Reverse primer
Human *BEX1*	5′-AGCAAGGAACGGGAAATAGA-3′	5′-CAGGAGAACACCCAGTTTAGAG-3′
Human *BEX4*	5′- GATACAGCCGCCCTCTTTT-3′	5′-GCCCGTCTTCCAGTTCATAG-3′
Human *CCN1*	5′-AGATCAAGGCAGTCGCTCTG-3′	5′-AAAGCACAGTCTTCCTCCGA-3′
Human *CCN2*	5′-AAAGTTGTGTAAGATTCACCAGCCT-3′	5'-GGTCGTTTACTCTCCATGACATCA-3'
Human *GAPDH*	5'-ATGACATCAAGAAGGTGGTG-3'	5'-CATACCAGGAAATGAGCTTG-3'

Supplemental Table S2. Sequence of BEX1 and BEX4 siRNAs.

siRNA	Sequence
BEX1 siRNA	5'-CUGCUUUCGAAUUUACAUG-3'
BEX4 siRNA	5'-GACCUAUCGUCCAGACUCA-3'

Supplemental Materials and methods

Immunohistochemistry (IHC)

IHC was conducted as described previously[1]. Brain samples were embedded in paraffin, and tissue sections were prepared using HistoCore AutoCut (Leica, Deerfield, IL, USA). Next, the sections were treated with 3% hydrogen peroxide/methanol and with 0.25% pepsin to retrieve antigens. The samples were incubated in blocking solution (Dako, Carpinteria, CA, USA), and were incubated at 4 °C overnight with the primary antibodies diluted in the antibody diluent (Dako). Then the sections were washed with Tris-buffered saline with 0.1% Tween 20 and incubated with a polymer-horseradish peroxidase-conjugated secondary antibody (Dako). A 3,3′-diaminobenzidine substrate chromogen system (Dako) was used to detect antibody binding. Stained sections were visualized with an Olympus IX71 inverted microscope (Olympus Optical, Tokyo, Japan).

Clonogenic assay

A clonogenic assay to assess cell viability and proliferation after treatment was performed as described in a previous study[2]. Cells were seeded at a density of 500 cells in 35-mm dishes, and 24 h later, they were treated with siRNAs for BEX1, BEX4, or Lat B. After 24 h of treatment, the cells were grown at 37 °C in a 5% CO_2/95% air atmosphere for 14 d. Next, the cells were fixed with 10% methanol/10% acetic acid, stained with 1% crystal violet, and scanned for data acquisition.

Total RNA isolation and qRT-PCR

For mRNA expression assessment, qRT-PCR was performed as described previously[3]. (Applied Biosystems, Foster City, CA). It was performed for 40 cycles at 95°C for 15 s and 60°C for 1 min, followed by thermal denaturation. The primer sequences used are listed in Supplementary Table S1.

Western blot analysis

Protein expression was measured as described previously[4]. B riefly, whole cell lysates (WCL) were prepared using radioimmun oprecipitation assay (RIPA) lysis buffer (50 mM Tris, pH 7.4, 150 mM NaCl, 1% Triton X-100, 25 mM NaF, 1 mM dithiothreitol (D TT), and 20 mM EGTA supplemented with protease inhibitors), a nd the protein concentrations were measured using a Bio-Rad pr otein assay kit (Bio-Rad Laboratories, Hercules, CA). Protein sam ples were subjected to SDS-PAGE, transferred to a nitrocellulose membrane, and blocked with 5% bovine serum albumin in TBST (10 mM Tris, 100 mM NaCl, and 0.1% Tween 20). Next, membra nes were probed with primary antibodies and peroxidase-conjuga ted secondary antibodies (Santa Cruz Biotechnology, Santa Cruz, CA). The membranes were analyzed using an ECL detection syst em (Roche Applied Science, Indianapolis, IN) with iBright chemi-doc fl000 from Thermo Fisher Scientific.

References

1. 1. Son B, Kwon T, Lee S, Han I, Kim W, Youn H, *et al.* CYP2E1 regulates the development of radiationinduced pulmonary fibrosis via ER stress- and ROS-dependent mechanisms. *Am J Physiol Lung Cell Mol Physiol* 2017, **313**(5)**:** L916-l929.
2. 2. Kim W, Youn H, Kang C, Youn B. Inflammation-induced radioresistance is mediated by ROS-dependent inactivation of protein phosphatase 1 in non-small cell lung cancer cells. *Apoptosis* 2015, **20**(9)**:** 1242-1252.
3. 3. Son B, Lee S, Kim H, Kang H, Jeon J, Jo S, *et al.* Decreased FBP1 expression rewires metabolic processes affecting aggressiveness of glioblastoma. *Oncogene* 2020, **39**(1)**:** 36-49.
4. 4. Son B, Lee S, Kim H, Kang H, Kim J, Youn H, *et al.* Low dose radiation attenuates inflammation and promotes wound healing in a mouse burn model. *J Dermatol Sci* 2019, **96**(2)**:** 81-89.
